# Association of the time interval between first and last birth with obesity in middle-aged postmenopausal Chinese women: a cross-sectional study in southern China

**DOI:** 10.3389/fmed.2025.1505319

**Published:** 2025-03-31

**Authors:** Zhenzhen Su, Yanfang Luo, Fen Ye, Jiahui Xu, Hui Lu, Lingyun Zhu

**Affiliations:** ^1^Department of Gastroenterology, Affiliated Hospital of Jiangnan University, Wuxi, Jiangsu, China; ^2^Department of Neurology, Affiliated Hospital of Jiangnan University, Wuxi, Jiangsu, China; ^3^Department of Oncology, Affiliated Hospital of Jiangnan University, Wuxi, Jiangsu, China

**Keywords:** birth interval, obesity, anthropometric measurements, childbirth, reproductive factors, Chinese

## Abstract

**Background:**

Birth interval is acknowledged as a significant factor affecting the health of women and their children. This study aimed to investigate the relationship between the time interval from the first to the last birth and the prevalence of general obesity, abdominal obesity, or both, among middle-aged postmenopausal Chinese women.

**Methods:**

This cross-sectional survey examined 4,799 Chinese postmenopausal women, aged 45–59 years, who had at least one live birth. General obesity was assessed using body mass index (BMI), while abdominal obesity was evaluated through waist-to-hip ratio (WHR), waist-to-height ratio (WHtR), and waist circumference (WC). Multivariate linear and logistic regression models were employed to analyze the associations between the time interval from the first to the last birth and obesity.

**Results:**

The values of all four obesity measures increased with a longer time interval between the first and last births (*P* for trend <0.001). After adjusting for potential confounding variables, women with an interval of 10 or more years between their first and last birth exhibited a prevalence of obesity that was 1.49 times (95% confidence interval [CI], 1.12–1.99) higher by BMI, 1.29 times (95% CI, 1.02–1.61) higher by WC, 1.23 times (95% CI, 1.04–1.69) higher by WHtR, and 1.50 times (95% CI, 1.01–2.12) higher by WHR when compared to those with a 0 to 1-year interval.

**Conclusion:**

The time interval between the first and last birth was positively associated with an increased risk of obesity, particularly abdominal obesity, in middle-aged postmenopausal Chinese women. Therefore, healthcare providers should prioritize reproductive health issues among women, actively promote appropriate birth intervals, and advocate for evidence-based pregnancy planning for women of childbearing age. Moreover, these research findings offer valuable scientific insights for policymakers, enabling them to develop more targeted obesity prevention and management strategies specifically tailored to this demographic group.

## 1 Introduction

Obesity emerged as a significant public health challenge globally, affecting approximately 604 million adults and constituting about 12% of the adult population worldwide (1). Recent epidemiological studies indicated that the obesity rate among Chinese adults had reached 12%, positioning China as the country with the highest total number of obese individuals (2, 3). These statistics highlighted that China, as a rapidly developing nation, was experiencing a notable increase in obesity rates.

It is widely accepted that a variety of reproductive factors influence a woman’s life continuum, from the development of the female reproductive system to the aging process (4). Numerous studies have demonstrated that obesity could adversely impact reproductive health, affecting women’s ovulation, menstrual function, fertility, infertility treatment success rates, and obstetric outcomes (5, 6). However, it remains uncertain whether the biological changes that occur during pregnancy, including hormonal adaptations and postpartum behaviors, influence the distribution pattern of adiposity (7). The bidirectional relationship between obesity and reproduction—particularly regarding how reproductive factors affect the outcomes of obesity in women—has emerged as a critical area of scientific inquiry.

Pregnancy, a pivotal aspect of the female reproductive cycle, is characterized by a myriad of complex physiological and psychological changes. Following the relaxation of China’s fertility policy, a noticeable increase in births among middle-aged women was observed, resulting in varying birth intervals (8, 9). Birth interval, defined as the length of time between two successive childbirths for the same woman, is crucial for safeguarding the health of both mothers and infants (10). Numerous studies have demonstrated that shorter birth intervals were associated with an elevated risk of maternal health complications, including cardiovascular and cerebrovascular diseases, as well as hypertension (11, 12). A cross-sectional study involving 1,045 Sudanese women concluded that longer intervals between births were linked to maternal obesity and overweight (13). Furthermore, a meta-analysis reported that weight gain during pregnancy correlates with postpartum weight retention and, in some instances, obesity (14). The prevalence of obesity related to childbearing has been on the rise among middle-aged and older women (15). However, much of the existing literature primarily focused on the impact of intervals between consecutive live births on obesity in women of reproductive age, often neglecting the importance of the interval between the first and last birth, which provides a broader temporal perspective (16). This oversight is particularly significant for middle-aged postmenopausal women, who have completed their reproductive years and may be affected by the cumulative effects of reproductive factors over time. Therefore, examining the relationship between the interval from the first to the last birth and obesity status in this specific population is essential for enhancing our understanding of the underlying causes of obesity and for developing effective intervention strategies.

Several anthropometric measurements, including body mass index (BMI), waist circumference (WC), waist-to-height ratio (WHtR), and waist-to-hip ratio (WHR), have been identified as commonly used to assess obesity (17). Each of these indicators possesses unique strengths and limitations. For instance, while BMI was widely recognized, it did not differentiate between fat and muscle mass (18). In contrast, WC and WHtR specifically evaluated fat accumulation in the abdominal area, whereas WHR assessed fat distribution in the hips (19). Recent evidence suggested that the relationship between birth intervals and obesity varied significantly across different cultures, ethnic groups, and developmental stages within countries (14). Despite this expanding body of literature, a significant gap in research remained, focusing on the association between obesity and the time interval between the first and last childbirth among Chinese women. This study aimed to examine whether the duration between the first and last birth was associated with general obesity, abdominal obesity, or both, particularly in middle-aged postmenopausal women in China. Through meticulous analysis of anthropometric indices, this study aimed to establish a scientific foundation for developing effective strategies to prevent overweight and obesity, while also promoting reproductive health among Chinese women.

## 2 Methods

### 2.1 Study design, period, and setting

This study was conducted in Jiangsu Province, located in southern China, from January 2022 to March 2023. The research was carried out by trained health workers using a community-based cross-sectional survey design, which recruited a representative sample of 4,799 participants. These participants included residents from both urban and rural communities across various cities within the province. Jiangsu Province, situated in eastern China (30°-35°N, 116°-121°E), spans approximately 102,600 square kilometers and features a diverse topography that encompasses plains, hills, and coastal regions. The province is composed of 13 prefecture-level cities, which are further subdivided into counties and districts, making Jiangsu one of the most economically advanced and densely populated provinces in China. To ensure a diverse and representative sample, participants were recruited through community health service centers, local community organizations, and residential areas.

### 2.2 Populations

The source population consisted of middle-aged Chinese women, aged between 45 and 59 years, who were naturally menopausal and had each experienced at least one live birth. Participants were selected through a stratified random sampling method. Exclusion criteria included women with specific conditions such as malignant tumors, mental disorders, a history of hysterectomy or oophorectomy, current use of oral contraceptives or hormone replacement therapy, and those lacking complete data on the time interval between their first and last birth or obesity measurements.

### 2.3 Ethical consideration

All participants provided informed written consent, and the study was approved by the medical ethics committee of the Affiliated Hospital of Jiangnan University. Participant anonymity was strictly maintained throughout the research process. This study adhered to the guidelines set forth in the Declaration of Helsinki, ensuring that only authorized personnel had access to the data. Data access rights were rigorously controlled. Furthermore, research data were securely stored in an encrypted database with a retention period of ten years post-study completion, as required by relevant regulations and the ethics committee. These measures ensured the confidentiality and integrity of the data.

### 2.4 Data collection instruments

Data collection was conducted by well-trained health workers. To enhance their investigative skills and data collection capabilities, unified training sessions and regular group discussions were implemented. Each participant was thoroughly informed about the study and required to complete a structured questionnaire that gathered information on sociodemographic characteristics, health-related variables, female reproductive factors, and physical measurements. Participants were instructed to complete the written questionnaires independently. For those facing challenges, such as difficulties in reading or writing, researchers provided assistance by asking questions orally to ensure comprehension and appropriate responses. All questionnaires were collected on-site, where researchers conducted a preliminary review. If any items were found to be incomplete, participants were prompted to supplement the missing information. Questionnaires with inconsistent answers or apparent systematic errors were deemed invalid. The estimated completion time for the questionnaire was approximately 20 min. Participants retained the right to withdraw from the study at any stage without facing penalties or adverse consequences. Upon completion of the written questionnaire, participants received a gift valued at 30 RMB as a token of appreciation.

### 2.5 Anthropometric measurements

Anthropometric measurements (height, weight, waist circumference [WC], hip circumference [HC]) were carried out using standard apparatus while participants were wearing light clothes without hats and shoes. Standing height was measured to the nearest 0.1 cm using a stadiometer, and weight was measured using an electronic body scale. WC and HC were both measured using a soft, non-stretchable tape. Specifically, WC was determined midway between the iliac crest and the lowest rib, and HC was measured as the largest circumference around the buttocks. The anthropometric indices were calculated as following: BMI = weight (kg)/ height^2^ (m^2^), WHtR = WC (cm)/ height (cm), WHR = WC (cm)/ HC (cm). Consistent with previous studies, general obesity was defined as BMI ≥24.0 kg/m^2^ (including 24.0–27.9 kg/m^2^ for overweight and ≥ 28.0 kg/m^2^ for obese) and abdominal obesity was considered as WC ≥80.0 cm, both recommended by Chinese guidelines (20). Meanwhile, other cut-off points for abdominal obesity, such as WHR ≥ 0.85, were proposed by the WHO, and WHtR ≥0.5 was recommended by previous studies (21).

### 2.6 Study variables

This general study questionnaire was composed of questions on sociodemographic variables, health-related variables, female reproductive factors, and physical measurements, as illustrated in Table 1. Sociodemographic variables consisted of age, marital status, medical insurance, monthly household income, and education level. Health-related factors comprised smoking status, drinking status, sitting time, exercise, chronic diseases (including hypertension, diabetes mellitus, and dyslipidemia), and food habits (including vegetable/fruit intake, salty food preference, dairy product consumption, and taste preference). Female reproductive factors included the number of pregnancies, abortions, parity, mode of delivery, ever breastfeeding, and the age at first birth and last birth, which were self-reported by the respondents. Physical measurements included height, weight, WC, and HC. Specifically, exercise was measured with the question “How many times a week do you exercise on average?.” Sitting time was assessed based on the response to the question “How many hours do you sit still on an average day?” (22). The time interval between the first and last birth was calculated by subtracting the age at first birth from the age at last birth of the postmenopausal women (23). Natural menopause is characterized by the absence of menstrual bleeding for a minimum of 12 consecutive months, with no prior history of hysterectomy or oophorectomy (5).

**Table 1:** The descriptive characteristics of 4,799 middle-aged Chinese post-menopausal women by the time interval between first and last birth.

Variable	Time interval between first and last birth (years)				*χ^2^/F*	*P* for trend
	0-1 (*n* = 1,543)	2-4 (*n* = 1,553)	5-9 (*n* = 1,401)	≥10 (*n* = 302)		
Sociodemographic factors, % or mean (SD)						
Age (years)	50.78 ± 4.28	52.49 ± 4.06	52.28 ± 4.24	50.86 ± 4.53	55.037^**^	<0.001
Marital status					8.391^*^	0.039
Married	1,460 (31.75)	1,498 (32.57)	1,350 (29.35)	291 (6.33)		
Divorced/widowed	83 (41.50)	55 (27.50)	51 (25.50)	11 (5.50)		
Medical insurance					60.656^**^	<0.001
Yes	1,373 (32.93)	1,359 (32.59)	1,218 (29.21)	220 (5.28)		
No	170 (27.03)	194 (30.84)	183 (29.09)	82 (13.04)		
Monthly household income					21.225^*^	0.002
<3,000 RMB	630 (29.02)	741 (34.13)	665 (30.63)	135 (6.22)		
3,000–5,000 RMB	374 (35.12)	325 (30.52)	307 (28.83)	59 (5.54)		
>5,000 RMB	539 (34.48)	487 (31.16)	429 (27.45)	108 (6.91)		
Education level					213.944^**^	<0.001
Primary school or lower	464 (24.34)	685 (35.94)	619 (32.48)	138 (7.24)		
Middle school	693 (31.76)	717 (32.86)	641 (29.38)	131 (6.00)		
High school or above	386 (54.29)	151 (21.24)	141 (19.83)	33 (4.64)		
Health-related factors, %						
Current/Former smoker					21.953^**^	<0.001
Yes	9 (20.93)	14 (32.56)	10 (23.26)	10 (23.26)		
No	1,534 (32.25)	1,539 (32.36)	1,391 (29.25)	292 (6.14)		
Passive smoking					13.309^*^	0.004
Yes	551 (29.15)	644 (34.07)	576 (30.48)	119 (6.30)		
No	992 (34.10)	909 (31.25)	825 (28.36)	183 (6.29)		
Current/Former alcohol drinker					26.929^**^	<0.001
Yes	34 (25.56)	34 (25.56)	43 (32.33)	22 (16.54)		
No	1,509 (32.34)	1,519 (32.55)	1,358 (29.10)	280 (6.00)		
Sitting time					10.936^*^	0.012
<6 h	942 (30.77)	1,033 (33.75)	901 (29.43)	185 (6.04)		
≥6 h	601 (34.58)	520 (29.92)	500 (28.77)	117 (6.73)		
Exercise					37.212^**^	<0.001
≥3 time/week	817 (33.35)	787 (32.12)	724 (29.55)	122 (4.98)		
1–2 time/week	244 (38.13)	183 (28.59)	170 (26.56)	43 (6.72)		
No exercise	482 (28.20)	583 (34.11)	507 (29.67)	137 (8.02)		
Hypertension					16.702^*^	0.001
Yes	159 (26.68)	212 (35.57)	171 (28.69)	54 (9.06)		
No	1,384 (32.93)	1,341 (31.91)	1,230 (29.26)	248 (5.90)		
Diabetes Mellitus					14.767^*^	0.002
Yes	34 (22.52)	54 (35.76)	44 (29.14)	19 (12.58)		
No	1,509 (32.47)	1,499 (32.25)	1,357 (29.19)	283 (6.09)		
Dyslipidemia					15.506^*^	0.001
Yes	39 (25.66)	40 (26.32)	54 (35.53)	19 (12.50)		
No	1,504 (32.36)	1,513 (32.56)	1,347 (28.99)	283 (6.09)		
Vegetable/Fruit					15.931^*^	0.014
Never	76 (26.95)	106 (37.59)	73 (25.89)	27 (9.57)		
Sometimes	410 (31.98)	434 (33.85)	357 (27.85)	81 (6.32)		
Often	1,057 (32.67)	1,013 (31.31)	971 (30.02)	194 (6.00)		
Salty food					45.066^**^	<0.001
Never	503 (33.29)	487 (32.23)	429 (28.39)	92 (6.09)		
Sometimes	1,008 (32.10)	1,021 (32.52)	929 (29.59)	182 (5.80)		
Often	32 (21.62)	45 (30.41)	43 (29.05)	28 (18.92)		
Dairy product					41.732^**^	<0.001
Never	747 (28.99)	897 (34.81)	744 (28.87)	189 (7.33)		
Sometimes	466 (34.62)	399 (29.64)	416 (30.91)	65 (4.83)		
Often	330 (37.67)	257 (29.34)	241 (27.51)	48 (5.48)		
Taste preference					22.899^*^	0.006
Medium	866 (31.11)	949 (34.09)	814 (29.24)	155 (5.57)		
Salty	132 (30.70)	138 (32.09)	122 (28.37)	38 (8.84)		
Bland	528 (34.51)	447 (29.22)	453 (29.61)	102 (6.67)		
Oily	17 (30.91)	19 (34.55)	12 (21.82)	7 (12.73)		
Reproductive factors, % or mean (SD)						
Number of pregnancies	1.50 ± 0.77	2.25 ± 0.72	2.59 ± 0.91	2.72 ± 0.93	533.060^**^	<0.001
Number of abortion	0.23 ± 0.66	0.15 ± 0.56	0.18 ± 0.58	0.21 ± 0.58	4.739^*^	0.003
Parity	1.27 ± 0.46	2.10 ± 0.50	2.42 ± 0.75	2.50 ± 0.80	1085.688^**^	<0.001
Mode of delivery					58.379^**^	<0.001
Cesarean section	213 (39.96)	106 (19.89)	158 (29.64)	56 (10.51)		
Vaginal delivery	1,330 (31.18)	1,447 (33.92)	1,243 (29.14)	246 (5.77)		
Ever breastfeeding	1,479 (31.83)	1,522 (32.76)	1,354 (29.14)	291 (6.26)	12.382^*^	0.006
Age at first birth	26.65 ± 3.75	24.66 ± 2.69	24.33 ± 2.61	23.04 ± 2.76	218.503^**^	<0.001
Age at last birth	26.97 ± 3.70	27.67 ± 2.80	30.40 ± 2.77	35.22 ± 3.31	783.654^**^	<0.001

The numerical data are shown as mean ± standard deviation (SD) and evaluated using the F test; Categorical data are expressed as percentages in parentheses following the numbers and assessed with the chi-square (χ^2^) test. ^*^P < 0.05, ^**^P < 0.001.

### 2.7 Statistical analysis

Data analysis was conducted using SPSS version 21.0 (Chicago, IL, USA). Univariate, bivariate and multivariate analyses were performed in this study. The numerical data were summarized as means ± standard deviation (SD), while categorical variables were presented as frequencies and percentages. The differences among birth interval groups were tested using chi-square tests and analysis of variance (ANOVA), as appropriate. Additionally, we examined the impact and risk of increased birth interval on obesity using four hierarchical models in both logistic and linear regression. Specifically, Model 1 assessed the association between obesity and birth interval without adjusting for confounders. Model 2 adjusted for age, Model 3 further adjusted for parity, and Model 4 adjusted for the covariates in Model 3 plus marital status, medical insurance, monthly household income, education level, smoking status (current/former smoker, passive smoker), current/former alcohol drinker, sitting time, exercise, hypertension, diabetes mellitus, dyslipidemia, vegetable/fruit intake, salty food preference, dairy product consumption, taste preference, number of pregnancies, number of abortions, mode of delivery, and ever breastfeeding. To detect possible multi-collinearity in the model, the variance inflation factor (VIF) was calculated in general linear regression, while in logistic regression, results were presented as odds ratios (ORs) and 95% confidence intervals (95% CIs). A 2-tailed statistical significance level of *P* < 0.05 was applied for the analyses.

## 3 Results

### 3.1 Sociodemographic characteristics

The women’s average age was 51.78 ± 4.29 (SD) years. Women with longer time intervals between their first and last births were more likely to be less educated, have higher monthly household incomes, be current or former smokers and alcohol drinkers, and exercise less frequently. In addition, women with longer intervals between their first and last births tended to consume vegetables or fruits and dairy products less often. It was also found that the number of pregnancies, parity, and the prevalence of dyslipidemia showed an increasing trend with longer time intervals between first and last births. More detailed sample characteristics are exhibited in Table 1.

### 3.2 Trends in obesity measurements across birth intervals

**Figure 1:**
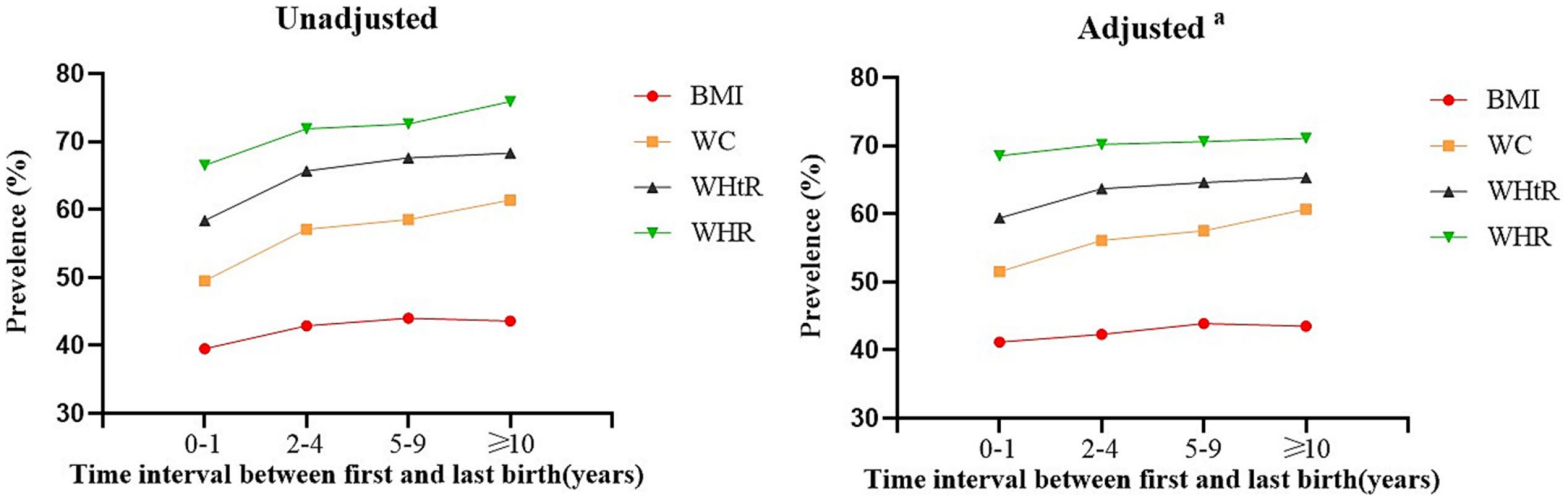
The prevalence of obesity stratified by the time interval between first and last birth with different anthropometric measures. BMI, body mass index; WC, waist circumference; WHtR, waist-to-height ratio; WHR, waist-to-hip ratio. ^a^Adjusted for age, parity, marital status, medical insurance, monthly household income, education level, smoking status (current/former smoker, passive smoker), current/former alcohol drinker, sitting time, exercise, hypertension, diabetes mellitus, dyslipidemia, vegetable/fruit, salty food, dairy product, taste preference, number of pregnancies, number of abortion, mode of delivery and ever breastfeeding.

Table 2 provides the mean values of the four obesity measurements after adjusting for age according to the time interval between first and last birth. With regard to the mean values of WC, BMI, WHtR, and WHR, an increasing trend with longer time intervals between first and last births was observed (*P* for trend <0.001). These four obesity measurements, irrespective of whether they were adjusted for confounders, all indicated that the prevalence of obesity was positively associated with the time interval between first and last births (Figure 1). Notably, WHR was associated with the highest prevalence of obesity both before and after adjustments, while the prevalence rate predicted by BMI was generally lower.

**Table 2:** Age-adjusted means (95% confidence intervals) of four measurements of obesity according to the time interval between first and last birth.

Measurement	Time interval between first and last birth (years)				*P* for trend
	0–1	2–4	5–9	≥ 10	
BMI (kg/m^2^)	23.04 (22.88–23.20)	23.70 (23.53–23.87)	24.02 (23.82–24.23)	24.78 (24.27–25.29)	<0.001
WC (cm)	79.64 (79.22–80.07)	81.21 (80.78–81.64)	81.61 (81.13–82.10)	82.75 (81.70–83.80)	<0.001
WHtR	0.510 (0.507–0.512)	0.521 (0.518–0.523)	0.523 (0.520–0.527)	0.529 (0.522–0.536)	<0.001
WHR	0.869 (0.866–0.872)	0.878 (0.875–0.881)	0.881 (0.877–0.884)	0.891 (0.883–0.900)	<0.001

BMI, body mass index; WC, waist circumference; WHtR, waist-to-height ratio; WHR, waist-to-hip ratio.

### 3.3 Linear regression analysis of obesity measurements

Linear regression analysis from the four models is shown in Table 3, in which the time interval between the first and last birth was assigned as a continuous variable. Generally, the VIF (Variance Inflation Factor) is used to judge whether there is multi-collinearity in the modeling process, and values of 10 or more are considered indicative of serious multi-collinearity. Regarding the results from this study, the VIF values of all variables did not exceed 2, indicating that multi-collinearity was unlikely in the model. Regression coefficients for the time interval between the first and last birth, considered as explanatory variables, were introduced into the four models successively. The results suggested that the four obesity measurements were, to some extent, significantly affected by the time interval between the first and last birth after adjusting for confounders (*P* < 0.05). Furthermore, the fully adjusted *β*-coefficients for the time interval between the first and last birth for WC, BMI, WHtR, and WHR were 0.41, 0.48, 0.0029, and 0.0042, respectively.

**Table 3:** β-coefficients (95% confidence intervals) for the time interval between first and last birth and different measurements of obesity.

	Model 1	Model 2	Model 3	Model 4
BMI	0.52 (0.41–0.63)	0.51 (0.40–0.62)	0.46 (0.33–0.60)	0.41 (0.27–0.54)
WC	0.99 (0.72–1.25)	0.89 (0.62–1.15)	0.60 (0.26–0.93)	0.48 (0.15–0.81)
WHtR^a^	0.66 (0.48–0.83)	0.57 (0.40–0.75)	0.39 (0.17–0.61)	0.29 (0.08–0.51)
WHR^a^	0.64 (0.45–0.84)	0.57 (0.37–0.76)	0.43 (0.19–0.68)	0.42 (0.17–0.66)
VIF for Time interval between first and last birth	1.00	1.58	1.67	1.82

BMI, body mass index; WC, waist circumference; WHtR, waist-to-height ratio; WHR, waist-to-hip ratio; VIF, variance inflation factor. ^a^β-coefficient amplified by 100. Model 1: unadjusted; Model 2: adjusted for age; Model 3: adjusted for covariate in Model 2 plus parity; Model 4: adjusted for covariates in Model 3 plus marital status, medical insurance, monthly household income, education level, smoking status (current/former smoker, passive smoker), current/former alcohol drinker, sitting time, exercise, hypertension, diabetes mellitus, dyslipidemia, vegetable/fruit, salty food, dairy product, taste preference, number of pregnancies, number of abortion, mode of delivery and ever breastfeeding.

### 3.4 Logistic regression analysis of obesity risk

Table 4 presents a summary of the results from the logistic regression analysis, in which the odds ratios and 95% confidence intervals for the time interval between the first and last birth with different measurements of obesity are available. Specifically, in the crude model, the time interval between the first and last birth was significantly associated with a higher prevalence of the four obesity measurements. In Model 2, we additionally adjusted for age and found that the OR values for the four obesity measurements decreased. In Model 3, we further adjusted for parity, and in this model, the OR values for the three abdominal obesity measurements decreased (WC, WHtR, and WHR), while the general obesity measurement (BMI) increased among the three birth interval groups. In Model 4, all variables were fully adjusted. Compared with a 0 to 1-year time interval between the first and last birth, an increasing risk of obesity was observed in women with at least a 10-year difference between their first and last childbirth, and the association between the time interval between the first and last birth and obesity was generally attenuated but remained statistically significant. For women who had an interval of 10 or more years between their first and last birth, the prevalence of obesity was 1.49 times higher by BMI, 1.29 times higher by WC, 1.23 times higher by WHtR, and 1.50 times higher by WHR when compared with those who had a 0 to 1-year interval. Our analysis, spanning from Model 1 to Model 4, revealed that a longer interval between a woman’s first and last birth is associated with a significant increase in the gradient of WC. In contrast, the gradients of BMI, WHtR, and WHR demonstrated a more stable trend over time, although these trends remained statistically significant.

**Table 4:** Odds ratios (95% confidence intervals) for the time interval between first and last birth with different measurements of obesity.

Measurement	Time interval between first and last birth (years)	Model 1	Model 2	Model 3	Model 4
BMI ≥ 24.0 kg/m^2^	0–1	1.00	1.00	1.00	1.00
	2–4	1.23 (1.06–1.42) ^*^	1.19 (1.05–1.38) ^*^	1.20 (1.04–1.42) ^*^	1.15 (1.01–1.36) ^*^
	5–9	1.39 (1.20–1.61) ^**^	1.36 (1.17–1.58) ^**^	1.37 (1.14–1.65) ^*^	1.34 (1.11–1.62) ^*^
	≥ 10	1.78 (1.39–2.28) ^**^	1.78 (1.39–2.28) ^**^	1.79 (1.36–2.36) ^**^	1.49 (1.12–1.99) ^*^
	*P* for trend	<0.001	0.001	0.001	0.007
WC ≥ 80 cm	0–1	1.00	1.00	1.00	1.00
	2–4	1.36 (1.18–1.57) ^**^	1.26 (1.09–1.46) ^*^	1.14 (0.96–1.34)	1.11 (0.94–1.31)
	5–9	1.44 (1.24–1.66) ^**^	1.35 (1.16–1.56) ^**^	1.16 (0.97–1.40)	1.13 (0.94–1.37)
	≥ 10	1.62 (1.29–2.09) ^**^	1.62 (1.29–2.09) ^**^	1.37 (1.07–1.82) ^*^	1.29 (1.02–1.61) ^*^
	*P* for trend	<0.001	<0.001	<0.001	0.001
WHtR ≥0.5	0–1	1.00	1.00	1.00	1.00
	2–4	1.45 (1.25–1.69) ^**^	1.32 (1.13–1.54) ^**^	1.21 (1.02–1.45) ^*^	1.17 (0.97–1.40)
	5–9	1.50 (1.28–1.75) ^**^	1.37 (1.18–1.62) ^**^	1.23 (1.01–1.50) ^*^	1.19 (0.97–1.46)
	≥ 10	1.66 (1.26–2.19) ^**^	1.67 (1.26–2.20) ^**^	1.47 (1.18–2.00) ^*^	1.23 (1.04–1.69) ^*^
	*P* for trend	<0.001	<0.001	<0.001	0.002
WHR ≥ 0.85	0–1	1.00	1.00	1.00	1.00
	2–4	1.28 (1.09–1.50) ^*^	1.19 (1.01–1.36) ^*^	1.10 (0.92–1.33)	1.09 (0.90–1.32)
	5–9	1.32 (1.12–1.56) ^*^	1.21 (1.02–1.43) ^*^	1.13 (0.92–1.40)	1.14 (0.92–1.41)
	≥ 10	1.67 (1.23–2.25) ^*^	1.66 (1.23–2.25) ^*^	1.54 (1.11–2.15) ^*^	1.50 (1.01–2.12) ^*^
	*P* for trend	<0.001	<0.001	<0.001	<0.001

BMI, body mass index; WC, waist circumference; WHtR, waist-to-height ratio; WHR, waist-to-hip ratio. ^*^P < 0.05, ^**^P < 0.001. Model 1: unadjusted; Model 2: adjusted for age; Model 3: adjusted for covariate in Model 2 plus parity; Model 4: adjusted for covariates in Model 3 plus marital status, medical insurance, monthly household income, education level, smoking status (current/former smoker, passive smoker), current/former alcohol drinker, sitting time, exercise, hypertension, diabetes mellitus, dyslipidemia, vegetable/fruit, salty food, dairy product, taste preference, number of pregnancies, number of abortion, mode of delivery and ever breastfeeding.

## 4 Discussion

This study conducted a comprehensive analysis of various anthropometric indicators, including BMI, WC, WHR, and WHtR. The primary objective was to investigate the relationship between the interval between the first and last births and the prevalence of obesity among Chinese middle-aged postmenopausal women. Our findings indicated that a longer interval between the first and last births was positively associated with an increased risk of obesity, particularly abdominal obesity.

Several studies have reported a positive correlation between birth intervals and weight gain (13–15). However, a few researches have produced conclusions that are inconsistent with the findings of this study. A retrospective cohort study conducted in Canada among a population of 38,178 women who had three or more deliveries found that a short interpregnancy interval was associated with an increased risk of gestational diabetes and the subsequent development of gestational obesity (24). Dewey et al. (25) also reported that shorter birth intervals (<12 months) may not provide adequate time for women to lose weight gained during the previous pregnancy. This discrepancy may arise from the fact that they focused on the interval between two consecutive live births, whereas our study examines the duration between the first and last birth, which encompasses a longer time span. Additionally, a longitudinal observational study involving 9,724 women indicated that the incidence of obesity increased with longer intervals between births (26). Concurrently, Mansour et al. (27) highlighted that among middle-aged Iraqis, a higher number of births was significantly associated with BMI and all three measures of abdominal obesity. This observation was consistent with the findings from those two studies that suggested a correlation between birth intervals and both general and abdominal adiposity within the Chinese population. Additionally, a systematic review and meta-analysis protocol suggested that both long and short birth intervals were linked to increased risks of adverse maternal, perinatal, obesity, and child health outcomes (28). Our study expanded on this understanding by demonstrating that the association between birth intervals and adiposity remained significant even after potential confounders such as age and parity had been controlled for. Furthermore, our results indicated that women with lower educational attainment and higher income levels tended to experience longer intervals between their first and last births, which was consistent with previous findings (29–31). This phenomenon could be attributed to the fact that lower educational levels often correlated with reduced health awareness and less informed reproductive choices, resulting in suboptimal birth spacing. Conversely, it was likely that women with higher incomes aimed to create better economic conditions for their children and may have been more inclined to delay childbirth, thereby increasing the spacing between births.

This study utilized various anthropometric indicators, including BMI, WC, WHR and WHtR to evaluate obesity in middle-aged postmenopausal women. Our findings indicated that WHR exhibited the strongest association with the interval between the first and last birth, emphasizing the pivotal role of abdominal adiposity in this specific population group. A population-based study among 7,771 women aged 25–64 years reported a generally positive correlation between parity and both BMI and WC, noting that visceral obesity was more prevalent among multiparous women compared to other groups (32). Conversely, a cross-sectional survey of 12,829 middle-aged and elderly Chinese women illuminated that, beyond a certain threshold of childbirths, BMI demonstrated a negative correlation with obesity risk, whereas markers of abdominal obesity like WHtR and WHR retained a persistent positive association over time (7). These two studies concurred with most of our results, suggesting that as the number of parities increases, so does the interval between the first and last birth, potentially leading to the accumulation of visceral adipose tissue without a corresponding increase in overall body fat. Pregnancy-induced insulin resistance has been shown to result in the deposition of triglycerides in visceral adipose tissue, which contributes to an increase in waist circumference (33, 34). Abdominal obesity, as compared to general obesity, exhibited a stronger correlation with insulin resistance. Notably, abdominal obesity is associated with a heightened risk of several metabolic diseases, including cardiovascular disease, diabetes, and metabolic syndrome (35). Consequently, for middle-aged postmenopausal women, indicators that reflect abdominal fat accumulation, such as WHR and WC, may offer enhanced diagnostic utility for chronic disease risk assessment.

Convincing evidence has indicated that the length of birth intervals is influenced by a multitude of factors, ranging from macro-level determinants such as policy and socioeconomic conditions to individual-level attributes including age, occupation, education, mobility, history of abortion, and contraceptive use (36, 37). It has been proven that the relationship between birth interval and female obesity is influenced by a plethora of factors (38). From a biological perspective, the risk of obesity could be influenced by birth interval, given its impact on female hormone levels and metabolic processes (39). It has been further indicated that the processes of labor and delivery may alter estrogen and progesterone levels, thereby reshaping a woman’s hormonal environment (40). These hormones, which play a crucial role in regulating fat distribution, energy metabolism, and appetite control, were found to be significantly affected by these physiological events (41). Concurrently, research has indicated that as women age, there is a gradual decline in ovarian function, resulting in decreased estrogen levels (42). This decline was associated with a reduced metabolic rate and a slowdown in fat burning, ultimately increasing the risk of obesity. Given that this physiological change may vary across different birth intervals, the specific underlying mechanism remains unclear. Apart from biological mechanisms, social and behavioral factors are also significantly implicated in shaping the relationship between birth interval and obesity. For instance, as women age, they tend to adopt a lifestyle characterized by higher calorie intake and lower physical activity, thereby passively increasing their risk of obesity (43). Furthermore, women who experience longer intervals between their first and last births may be subjected to heightened psychological stress, which can contribute to disordered lifestyle habits and, in turn, elevate their risk of obesity (44).

The rapid transformation of China’s population and economy is expected to intensify the burden of obesity-related diseases, particularly among middle-aged women, where this issue is anticipated to become increasingly pronounced. Our findings highlight the importance of addressing the cumulative effects of reproductive factors, such as birth intervals, on obesity risk. Therefore, it is crucial to develop targeted interventions aimed at preventing obesity and enhancing women’s reproductive health. This study provided a solid foundation for medical institutions and community health service centers to formulate specific intervention strategies. Specifically, it advocates leveraging online multimedia platforms to deliver health education tailored to middle-aged women, emphasizing the significance of appropriate birth intervals and their long-term health implications. Additionally, personalized pre-pregnancy counseling and fertility planning should be provided, taking into account the unique circumstances of women of childbearing age. Nutritional guidance, lifestyle advice, and postpartum health management should also be included, with particular emphasis on the physiological characteristics of middle-aged women, focusing on long-term support for weight management and chronic disease prevention among middle-aged postmenopausal women. Moreover, this study serves as a scientific reference for health policymakers. It is recommended that public awareness of health issues affecting middle-aged women be increased through media outreach and public health initiatives, while fostering a supportive social environment. Strengthening collaboration among medical institutions, community organizations, and government bodies is essential to establish a comprehensive health monitoring system, regularly evaluate intervention effectiveness, adjust strategies as necessary, and collectively advance the successful implementation of health intervention programs for middle-aged women.

Our study has several advantages over previous research on birth intervals and obesity. Firstly, to our knowledge, this is the first study to offer an in-depth analysis of the interval between the first and last births in relation to obesity among a large cohort of middle-aged postmenopausal Chinese women. Secondly, our study incorporates one general obesity measurement and three abdominal obesity measurements, each of which may possess distinct strengths in predicting risks for various comorbidities. Furthermore, all anthropometric measurements were conducted by trained professionals following standardized procedures, which greatly reduces the potential for measurement bias. Ultimately, this study benefited from a large sample size and extensive data on demographics, lifestyle factors, and reproduction-related variables, which not only enhanced the precision of our findings but also permitted robust statistical adjustments across multiple variables.

### 4.1 Limitations

This study provides valuable insights into the relationship between the first and last birth intervals and obesity among middle-aged postmenopausal Chinese women. However, several limitations should be acknowledged. First, the data were derived from participants’ self-reports, which may introduce reporting bias. Secondly, this study included women with at least one live birth but did not exclude those with only one child, which may potentially affect the accuracy of the interval estimates presented in the results. Due to its cross-sectional design, the study cannot establish a causal relationship between these birth intervals and obesity. Furthermore, the study population was confined to a specific region in China, which may limit the generalizability of the findings to other populations with different cultural and socioeconomic contexts. Future research should utilize longitudinal designs to ascertain the causal relationship between the first-to-last birth interval and obesity, as well as to investigate potential biological and sociological mechanisms.

## 5 Conclusion

In conclusion, our study found a significant positive correlation between a longer interval between the first and last births and an increased risk of obesity among middle-aged postmenopausal Chinese women, with a stronger association observed for abdominal obesity compared to general obesity. The findings of this study might highlight the necessity for health professionals to pay greater attention to the reproductive health and postpartum recovery of middle-aged women. Additionally, these insights may assist health policymakers in formulating targeted strategies for the prevention and control of obesity among middle-aged postmenopausal women.

## Data Availability

The original contributions presented in the study are included in the article/supplementary material, further inquiries can be directed to the corresponding authors.

## References

[1] Afshin A Forouzanfar MH Reitsma MB Sur P Estep K Lee A. Health effects of overweight and obesity in 195 countries over 25 years. N Engl J Med. (2017) 377:13–27. doi: 10.1056/NEJMoa1614362, PMID: 28604169 PMC5477817

[2] Pan XF Wang L Pan A. Epidemiology and determinants of obesity in China. Lancet Diabetes Endocrinol. (2021) 9:373–92. doi: 10.1016/s2213-8587(21)00045-0, PMID: 34022156

[3] Wang Y Zhao L Gao L Pan A Xue H. Health policy and public health implications of obesity in China. Lancet Diabetes Endocrinol. (2021) 9:446–61. doi: 10.1016/s2213-8587(21)00118-2, PMID: 34097869

[4] Aychiluhm SB Tadesse AW Mare KU Abdu M Ketema A. A multilevel analysis of short birth interval and its determinants among reproductive age women in developing regions of Ethiopia. PLoS One. (2020) 15:e0237602. doi: 10.1371/journal.pone.0237602, PMID: 32845940 PMC7449410

[5] Xu B Chen Y Xiong J Lu N Tan X. Association of Female Reproductive Factors with hypertension, diabetes and LQTc in Chinese women. Sci Rep. (2017) 7:42803. doi: 10.1038/srep42803, PMID: 28211485 PMC5314360

[6] Practice Committee of the American Society for Reproductive Medicine. Obesity and reproduction: a committee opinion. Fertil Steril. (2021) 116:1266–85. doi: 10.1016/j.fertnstert.2021.08.018, PMID: 34583840

[7] Li W Wang Y Shen L Song L Li H Liu B. Association between parity and obesity patterns in a middle-aged and older Chinese population: a cross-sectional analysis in the Tongji-Dongfeng cohort study. Nutr Metab (Lond). (2016) 13:72. doi: 10.1186/s12986-016-0133-7, PMID: 27795732 PMC5081958

[8] Yang Y He R Zhang N Li L. Second-child fertility intentions among urban women in China: a systematic review and Meta-analysis. Int J Environ Res Public Health. (2023) 20:3744. doi: 10.3390/ijerph20043744, PMID: 36834437 PMC9962327

[9] Chen Q Wang A Song X Liu X Liu Y Wei J. Fertility intentions to have a second or third child among the childbearing-age population in Central China under China's three-child policy: a cross-sectional study. J Glob Health. (2023) 13:04072. doi: 10.7189/jogh.13.04072, PMID: 37448328 PMC10345887

[10] Shifti DM Chojenta C Holliday EG Loxton D. Application of geographically weighted regression analysis to assess predictors of short birth interval hot spots in Ethiopia. PLoS One. (2020) 15:e0233790. doi: 10.1371/journal.pone.0233790, PMID: 32470020 PMC7259714

[11] Katuwal S Tapanainen JS Pukkala E Kauppila A. The effect of length of birth interval on the risk of breast cancer by subtype in grand multiparous women. BMC Cancer. (2019) 19:199. doi: 10.1186/s12885-019-5404-z, PMID: 30832620 PMC6399864

[12] Gebrehiwot SW Abera G Tesfay K Tilahun W. Short birth interval and associated factors among women of child bearing age in northern Ethiopia, 2016. BMC Womens Health. (2019) 19:85. doi: 10.1186/s12905-019-0776-4, PMID: 31266479 PMC6604155

[13] Ali EA Almugabil A Salim A Rayis DA Adam I. The effect of interpregnancy interval on obesity/overweight among women in the first trimester of pregnancy. Int J Gynaecol Obstet. (2017) 138:320–4. doi: 10.1002/ijgo.12222, PMID: 28555840

[14] Ni W Gao X Su X Cai J Zhang S Zheng L. Birth spacing and risk of adverse pregnancy and birth outcomes: a systematic review and dose-response meta-analysis. Acta Obstet Gynecol Scand. (2023) 102:1618–33. doi: 10.1111/aogs.14648, PMID: 37675816 PMC10619614

[15] Devakumar D Hallal PC Horta BL Barros FC Wells JC. Association between birth interval and cardiovascular outcomes at 30 years of age: a prospective cohort study from Brazil. PLoS One. (2016) 11:e0149054. doi: 10.1371/journal.pone.0149054, PMID: 26890250 PMC4758625

[16] Wong TS Chay WY Tan MH Chow KY Lim WY. Reproductive factors, obesity and risk of colorectal cancer in a cohort of Asian women. Cancer Epidemiol. (2019) 58:33–43. doi: 10.1016/j.canep.2018.10.016, PMID: 30448606

[17] Jayedi A Soltani S Motlagh SZ Emadi A Shahinfar H Moosavi H. Anthropometric and adiposity indicators and risk of type 2 diabetes: systematic review and dose-response meta-analysis of cohort studies. BMJ. (2022) 376:e067516. doi: 10.1136/bmj-2021-067516, PMID: 35042741 PMC8764578

[18] Geng S Chen X Shi Z Bai K Shi S. Association of anthropometric indices with the development of multimorbidity in middle-aged and older adults: a retrospective cohort study. PLoS One. (2022) 17:e0276216. doi: 10.1371/journal.pone.0276216, PMID: 36240163 PMC9565419

[19] Lima Borges L Rodrigues de Lima T Augusto Santos Silva D. Accuracy of anthropometric indicators of obesity to identify high blood pressure in adolescents-systematic review. PeerJ. (2022) 10:e13590. doi: 10.7717/peerj.13590, PMID: 35966930 PMC9373973

[20] Department of Medical Administration, National Health Commission of the People’s Republic of China. Guideline for diagnosis and treatment of obesity (2024th edition). Chin J Dig Surg. (2024):1237–60. doi: 10.3760/cma.j.cn115610-20241017-00455

[21] Nishida C Ko GT Kumanyika S. Body fat distribution and noncommunicable diseases in populations: overview of the 2008 WHO expert consultation on waist circumference and waist-hip ratio. Eur J Clin Nutr. (2010) 64:2–5. doi: 10.1038/ejcn.2009.139, PMID: 19935820

[22] Wu X Li B Lin WQ Huang LL Wang XX Fu LY. The association between obesity indices and hypertension: which index is the most notable indicator of hypertension in different age groups stratified by sex? Clin Exp Hypertens. (2019) 41:373–80. doi: 10.1080/10641963.2018.1489546, PMID: 30095294

[23] Bevier M Sundquist J Hemminki K. Does the time interval between first and last birth influence the risk of endometrial and ovarian cancer? Eur J Cancer. (2011) 47:586–91. doi: 10.1016/j.ejca.2010.10.004, PMID: 21055917

[24] Hanley GE Hutcheon JA Kinniburgh BA Lee L. Interpregnancy interval and adverse pregnancy outcomes: an analysis of successive pregnancies. Obstet Gynecol. (2017) 129:408–15. doi: 10.1097/aog.0000000000001891, PMID: 28178044

[25] Dewey KG Cohen RJ. Does birth spacing affect maternal or child nutritional status? A systematic literature review. Matern Child Nutr. (2007) 3:151–73. doi: 10.1111/j.1740-8709.2007.00092.x, PMID: 17539885 PMC6860904

[26] Reynolds CME Egan B O'Malley EG McMahon L Sheehan SR Turner MJ. Longitudinal study of maternal BMI in successive pregnancies. Obesity. (2020) 28:460–7. doi: 10.1002/oby.22707, PMID: 31970915

[27] Mansour AA Ajeel NA. Parity is associated with increased waist circumference and other anthropometric indices of obesity. Eat Weight Disord. (2009) 14:e50–5. doi: 10.1007/bf03327800, PMID: 19934637

[28] Shifti DM Chojenta C Hassen TA Harris ML. Short birth interval prevalence, determinants and effects on maternal and child health outcomes in Asia-Pacific region: a systematic review and meta-analysis protocol. BMJ Open. (2023) 13:e076908. doi: 10.1136/bmjopen-2023-076908, PMID: 38154890 PMC10759081

[29] Gurmu L Wakgari N Kolola T Danusa KT. Effect of short inter-pregnancy interval on perinatal outcomes among pregnant women in north-West Ethiopia: a prospective cohort study. Front Public Health. (2022) 10:953481. doi: 10.3389/fpubh.2022.953481, PMID: 36003632 PMC9393389

[30] Thoma ME Copen CE Kirmeyer SE. Short interpregnancy intervals in 2014: differences by maternal demographic characteristics. NCHS Data Brief. (2016):1–8. 27111053

[31] Panther E Amherdt S Macbeth M McNellis B Pan A Palatnik A. Incidence of adverse pregnancy outcomes based on the degree of short Interpregnancy interval in urban Milwaukee population. Wmj. (2023) 122:90–4. PMID: 37141470

[32] Luoto R Männistö S Raitanen J. Ten-year change in the association between obesity and parity: results from the national FINRISK population study. Gend Med. (2011) 8:399–406. doi: 10.1016/j.genm.2011.11.003, PMID: 22153883

[33] Creanga AA Catalano PM Bateman BT. Obesity in pregnancy. N Engl J Med. (2022) 387:248–59. doi: 10.1056/NEJMra1801040, PMID: 35857661

[34] Langley-Evans SC Pearce J Ellis S. Overweight, obesity and excessive weight gain in pregnancy as risk factors for adverse pregnancy outcomes: a narrative review. J Hum Nutr Diet. (2022) 35:250–64. doi: 10.1111/jhn.12999, PMID: 35239212 PMC9311414

[35] Yan Y Lu H Lin S Zheng Y. Reproductive factors and risk of cardiovascular diseases and all-cause and cardiovascular mortality in American women: NHANES 2003–2018. BMC Womens Health. (2024) 24:222. doi: 10.1186/s12905-024-03055-6, PMID: 38581038 PMC10996084

[36] Liu C Snowden JM Lyell DJ Wall-Wieler E Abrams B Kan P. Interpregnancy interval and subsequent severe maternal morbidity: a 16-year population-based study from California. Am J Epidemiol. (2021) 190:1034–46. doi: 10.1093/aje/kwab020, PMID: 33543241 PMC8168254

[37] Taylor RAM Yang JM Cheney K Black K. Short interpregnancy interval: circumstance or choice? BMJ Sex Reprod Health. (2022) 48:110–6. doi: 10.1136/bmjsrh-2021-201269 34649962

[38] Yong W Wang J Leng Y Li L Wang H. Role of obesity in female reproduction. Int J Med Sci. (2023) 20:366–75. doi: 10.7150/ijms.80189, PMID: 36860674 PMC9969507

[39] Liu W Ren L Fang F Chen R. Maternal pre-pregnancy overweight or obesity and risk of birth defects in offspring: population-based cohort study. Acta Obstet Gynecol Scand. (2024) 103:862–72. doi: 10.1111/aogs.14786, PMID: 38282287 PMC11019515

[40] Olza I Uvnas-Moberg K Ekström-Bergström A Leahy-Warren P Karlsdottir SI Nieuwenhuijze M. Birth as a neuro-psycho-social event: an integrative model of maternal experiences and their relation to neurohormonal events during childbirth. PLoS One. (2020) 15:e0230992. doi: 10.1371/journal.pone.0230992, PMID: 32722725 PMC7386571

[41] Uvnäs-Moberg K. The physiology and pharmacology of oxytocin in labor and in the peripartum period. Am J Obstet Gynecol. (2024) 230:S740–58. doi: 10.1016/j.ajog.2023.04.011, PMID: 38462255

[42] Leeners B Geary N Tobler PN Asarian L. Ovarian hormones and obesity. Hum Reprod Update. (2017) 23:300–21. doi: 10.1093/humupd/dmw045, PMID: 28333235 PMC5850121

[43] Mason C de Dieu TJ Duggan C Wang CY Alfano CM McTiernan A. Eating behaviors and weight loss outcomes in a 12-month randomized trial of diet and/or exercise intervention in postmenopausal women. Int J Behav Nutr Phys Act. (2019) 16:113. doi: 10.1186/s12966-019-0887-1, PMID: 31775800 PMC6882083

[44] Olsen NJ Larsen SC Rohde JF Stougaard M Händel MN Specht IO. Effects of the healthy start randomized intervention on psychological stress and sleep habits among obesity-susceptible healthy weight children and their parents. PLoS One. (2022) 17:e0264514. doi: 10.1371/journal.pone.0264514, PMID: 35271601 PMC8912262

